# Non-technical skills and teamwork in trauma: from the emergency department to the operating room

**DOI:** 10.3389/fmed.2023.1319990

**Published:** 2023-12-05

**Authors:** Henrique Alexandrino, Bárbara Martinho, Luís Ferreira, Sérgio Baptista

**Affiliations:** ^1^Faculty of Medicine, University of Coimbra, Coimbra, Portugal; ^2^Department of Surgery, Coimbra University Hospital Center, Coimbra, Portugal; ^3^Lusitanian Association for Trauma and Emergency Surgery, Coimbra, Portugal; ^4^Hospital Dr. Nélio Mendonça, Funchal, Madeira, Portugal; ^5^Medio Tejo Hospital Center, Tomar, Portugal

**Keywords:** trauma, non-technical skills, training, human factors, emergency room, operating room

## Abstract

Management of a trauma patient is a challenging process. Swift and accurate clinical assessment is required and time-sensitive decisions and life-saving procedures must be performed in an unstable patient. This requires a coordinated response by both the emergency room (ER) and operating room (OR) teams. However, a team of experts does not necessarily make an expert team. Root cause analysis of adverse events in surgery has shown that failures in coordination, planning, task management and particularly communication are the main causes for medical errors. While most research is focused on the ER trauma team, the trauma OR team also deserves attention. In fact, OR team dynamics may resemble more the ER team than the elective OR team. ER and OR trauma teams assemble on short notice, and their members, who are from different specialties and backgrounds, may not train regularly together or even know each other beforehand. And yet, they have to perform high-risk procedures and make high stake decisions, in a time-sensitive manner. The airline industry has long recognized the role of team training and non-technical skills (NTS) in reducing hazards. The implementation of the so called crew resource management or crisis resource management (CRM) has significantly made airline travel safer and the transposition to the medical context, with specific training in non-technical skills, has also brought great benefits. In fact, it is clear that adoption of non-technical skills (NTS) in healthcare has led to an increase in patient safety. In this narrative review we recapitulate some of the key non-technical skills and their relevance in trauma, with a focus on both the emergency department (ER) and the operating room (OR) teams, as well as on the transition of care from one to the other. Also, we explore the use of debriefing the team, as well as the roles of NTS training in both undergraduate and postgraduate settings. We review some of the existing trauma training courses and their roles in developing NTS. Finally, we briefly address the challenges posed by the development of trauma hybrid operating rooms.

“*In crisis we do not rise to the level of our expectations, we fall to the level of our training*”. Archilochus, Greek poet and soldier, *circa* 645 BCE

## Introduction

Management of a trauma patient is a challenging process. Swift and accurate clinical assessment is required, time-sensitive decisions and life-saving procedures must be performed in an unstable patient, often with incomplete information and under intense time pressure. This requires a coordinated response by both the emergency room (ER) and operating room (OR) teams. These teams are multidisciplinary, consisting of doctors of distinct specialties and nurses, all of whom are highly motivated to excel at their technical and decision-making skills. Usually, all have previously attended training programs in order to acquire and develop these individual skills.

However, a team of experts does not necessarily make an expert team. Root cause analysis of adverse events in surgery has shown that failures in coordination, planning, task management and particularly communication are the main causes for medical errors (1). Moreover, transitions in healthcare are fraught with mishaps in communication (2) and several studies have confirmed that most severe errors in trauma resuscitation are related to failures in communication (3, 4).

While most research is focused on the ER trauma team, the trauma OR team also deserves attention. In fact, OR team dynamics may resemble more the ER team than the elective OR team. ER and OR trauma teams assemble on short notice, and their members, who are from different specialties and backgrounds, may not train regularly together or even know each other beforehand. And yet, they have to perform high-risk procedures and make high stake decisions, in a time-sensitive manner. Thus, the stage is set for “a perfect storm” of errors and poor outcomes (5).

The airline industry has long recognized the role of team training and non-technical skills (NTS) in reducing hazards. The implementation of the so called crew resource management or crisis resource management (CRM) has significantly made airline travel safer and the transposition to the medical context, with specific training in non-technical skills, has also brought great benefits (6). In fact, it is clear that adoption of NTS in healthcare has led to an increase in patient safety (7).

In this narrative review we recapitulate some of the key non-technical skills and their relevance in trauma, with a focus on both the emergency department (ER) and the operating room (OR) teams, as well as on the transition of care from one to the other. Also, we explore the use of debriefing the team, as well as the roles of NTS training in both undergraduate and postgraduate settings. We review some of the existing trauma training courses and their roles in developing NTS. Finally, we briefly address the challenges posed by the development of trauma hybrid operating rooms.

## Non-technical skills in trauma

Non-technical skills (NTS) are social and cognitive skills that interfere with task performance and completion (8). Either in the ER or the OR context, proper team function requires that all team members, and particularly the team leader, should not only be knowledgeable and proficient in their clinical and technical skills, but also have a clear understanding of the critical role of NTS.

There are several NTS particularly relevant for trauma management:

- Situational awareness
- Role allocation
- Decision-making
- Leadership
- Communication

### Situational awareness

Situational awareness is defined as “*the perception of elements in the environment within a volume of time and space, the comprehension of their meaning and the projection of their status in the near future*” (9).

This means that the practitioner, usually the team leader, should go beyond the immediately available information, integrate all current data with previous knowledge and expand the consciousness both in physical space and in time. This is considered one of the most important NTS and usually requires some degree of clinical experience and previous exposure to similar situations. However, with adequate simulation-based training it can also be developed by the junior trainee (10).

Situational awareness starts immediately with prehospital notification. The prehospital notification is ideally provided using the AT-MIST (Age, Time, Mechanism, Injuries, Signs, Treatment) mnemonic. Although only indicative, it can provide a glimpse of the potential clinical status of the patient and likely needs, such as activating the massive hemorrhage protocol and other resources, such as the trauma OR. A trigger for damage control surgery can start at this stage.

After patient arrival and during primary survey, situation awareness is also required during the assessment of the patient’s response to resuscitation. The availability of resources, physical and human, as well as the environment, come into play when deciding the predicted course of action. An example of the proper use of situational awareness is the branching decision process taking place inside the team leader’s mind well ahead of the information required to take the decision being available. For instance, immediate transfer to the OR vs. further resuscitation while an extended focused assessment sonogram in trauma – eFAST exam is ordered; laparotomy if eFAST positive for peritoneal fluid vs. thoracotomy if positive eFAST for pericardial fluid. The patient may just be undergoing the eFAST scan and these scenarios and subsequent branching decisions are being processed by the trauma team leader well in advance.

While the team leader should maintain situational awareness at all times during the ER resuscitation, it may be difficult for all the team members to keep up. In fact, it may not be desirable, especially when some of them are performing technical procedures requiring focus vision. However, it is incumbent upon the team leader of the trauma ER to periodically share the status and plan with all team members. This can be achieved with a Stop procedure, or Team-Time-Out. When clinically possible, i.e., not interfering with immediate resuscitation efforts, this time-out can be useful to share situational awareness with all the team (11).

**Figure 1:**
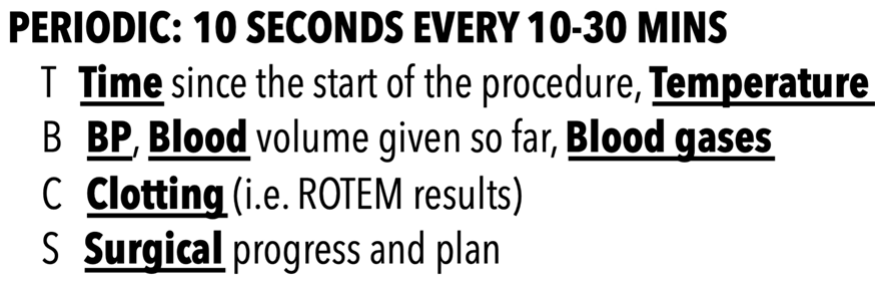
TBCS (Time, Blood, Clotting, Surgical) mnemonic for intraoperative time-outs in trauma damage control surgery.

Intraoperatively it can also be difficult for all team members to attain and maintain situational awareness. During the operation both the surgeon and the anesthesiologist will have to perform delicate procedures requiring focus vision, causing a potential decrease in situation awareness. Since there may be unexpected intraoperative adverse events it is paramount that situational awareness can alternate between the shared team leaders of the trauma team – surgeon and anesthesiologist. A tool to prevent this is the use of routine situation reports, or “sit reps” (12), actually a form of intraoperative Team-Time-Out. During these, provided there is temporary control of bleeding, the entire team briefs with a concise update. A useful structure for this is the TBCS (Time, Blood, Clotting, Surgical) mnemonic, first developed in the military advanced surgical hospitals, but easily transferable to the civilian setting (Figure 1).

The first three items of the TBCS refer to physiological variables and are reported by the anesthesiologist. The last item consists of surgical findings and plan and is reported by the surgeon. By using the TBCS tool, the entire trauma OR team can be regularly updated, and situational awareness shared with the members.

Another option is for the lead surgeon to share situational awareness with an assistant surgeon. With a two-surgeon team, it is helpful that one of the two can maintain situational awareness, especially when the operating surgeon is on focus vision. Nonetheless, both surgeons’ attention may be required in the operative field at times, making the use of intraoperative timeouts essential to keep situational awareness. The same can also work in a two-anesthesiologists team, with one performing procedures and another maintaining situational awareness. However, this should not replace the regular use of intraoperative timeouts.

Finally, situational awareness should not only cover the patient, the resources and the context, but should also contemplate the team members. Some team members may be overwhelmed, while others may be unused. These may create a sense of disenchantment with trauma resuscitation that may compromise the current resuscitation efforts and future trauma scenarios. The team leader(s) should recognize and anticipate this, properly reallocating roles and assigning new tasks.

### Role allocation

Role allocation is critical in the ER setting, where the team can be particularly diverse (13). While the role of team leader is usually performed by either trauma surgeon or emergency physician (14), other team members can be flexibly allocated, according to their expertise and level of comfort. One example is the anesthesiologist, who by essence of training is extremely well suited to lead a team, can manage most airway scenarios, is proficient in analgesia and sedation, and is thus usually allocated the role of Airway (A) doctor. Moreover, the inclusion of the anesthesiologist in the ER trauma team has the added advantage of providing continuity of care should the patient require operative treatment.

**Figure 2:**
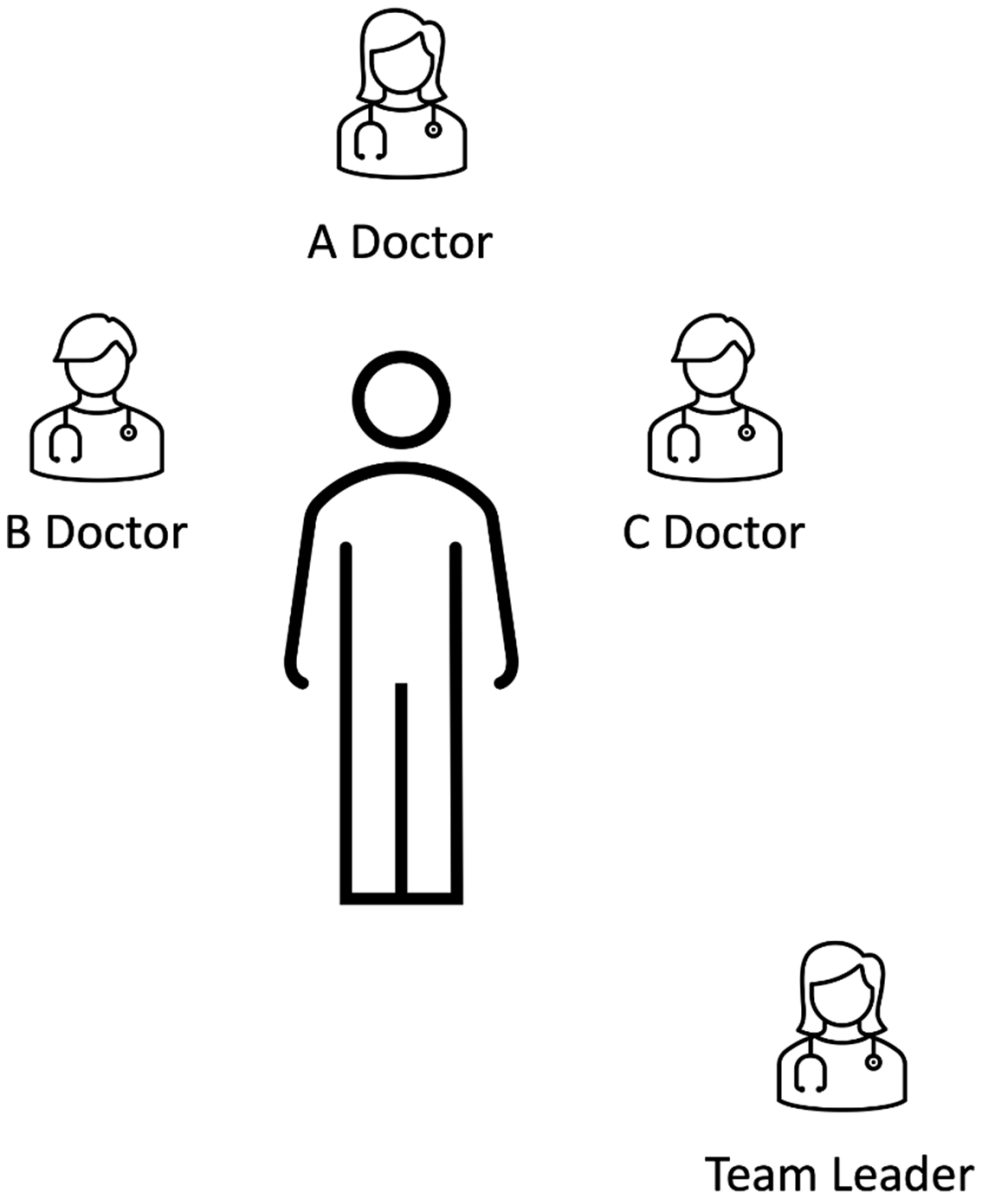
Typical role allocation of a trauma team according to the European Trauma Course. However, other options exist, depending on local protocols. Importantly, roles should be allocated and a team leader clearly designated.

Other trauma team roles are: the B (Breathing) doctor, assessing ventilation and performing thoracostomy and placement of chest drainage; the C (Circulation) doctor, assessing circulatory status, obtaining venous access and performing bleeding control procedures, such as application of pelvic binder (Figure 2). Multiple medical specialties are allocatable to these functions, with emergency medicine and trauma and emergency surgery obvious options. Clear allocation of ER nurses to each position is also desirable. In fact, a trauma team can be composed of sub-teams, each with its own responsibilities. Ultimately, all report to the team leader.

Role allocation may be dynamic. A good example is during airway management. While simple maneuvers can be provided by the A-doctor, drug-assisted intubation will require a reallocation of roles, for instance for drug administration, manual restriction of spinal movements and handing the laryngoscope and tube. The team leadership may even be temporarily handed over to the A-person, if he/she is an experienced provider. Other procedures requiring an assistant (such as placing a chest drain or a pelvic binder) will also require reassigning the team members to new tasks. Moreover, the team leader may have to briefly discuss or consult with another specialist and in order to prevent the loss of situational awareness, temporarily handover the leadership to another team member.

Allocation of roles within the team should adapt to local resources. In one of the authors’ institution trauma team training program, the simulations follow the composition of the trauma team, not vice versa. The motto that inspires the trauma team training program is: “We simulate like we work and work like we simulate.” A rigid team structure would be hard to follow and have little compliance, while a more realistic approach is expected to work much better.

While in the ER role allocation is flexible, with some members able to perform multiple tasks, it is usually more rigid and obvious in the OR: the surgeons will perform the operation, and the anesthesiologist will manage general anesthesia and resuscitation. However, some degree of role allocation is also required in the trauma OR, for instance for contacting the blood bank, placing lines, bringing extra equipment. A good moment to do this is during patient handover from the ER to the OR, when all the operative team should brief. The scrub and anesthesia nurses should have their roles clearly defined, and all team members’ names should be clearly identified. After this initial OR team briefing, the surgeon and anesthesiologist can brief their respective nursing staff with more detail (see below – Six-step approach to perioperative communication).

The surgeon’s role during induction also requires allocation, particularly because the patient may require a surgical airway, or a chest decompression for a tension pneumothorax after positive pressure ventilation. A member of the surgical team should be clearly allocated for this function, should the need arise.

While the best person should be designated to the proper position, in selected cases junior or more inexperienced team members may assume positions in order to provide proper exposure and training, and enable a rotation of functions. As usual, good judgement in balancing patients’ needs with training issues is mandatory. These members should be actively involved in the debriefing, to provide the maximum from the learning opportunity of participating in a trauma team (see below).

Role allocation, like most NTS, requires excellent communication between the team members.

### Decision-making

It is often said that good judgement comes with experience and that experience comes with bad judgement. Taking a neuroscience approach, we can divide the thought process used in decision making in two distinct pathways: type 1 and type 2. Type 1 decisions are based on pattern recognition, intuitive and require little mental effort. It is used for simple, daily tasks. Type 2 decisions use deductive processes, are logical, concept-based, require mental work and are thus slower (15).

The way we use these two processes in task performance is well exemplified when we walk in the street. While walking in the streets of our hometown we use mental shortcuts, with fast and automatic processes (type 1). However, when walking the streets of a foreign town (without an online map) we have to use deductive reasoning to find our way, taking considerably more time and mental effort (type 2).

Although we use the two decision-making processes interchangeably in our daily life, in stressful scenarios, our brain, for evolutionary reasons, is prompted to use type 1 decisions (15). A good example of the importance of the decision-making process is in the management of the bleeding patient. Both the experienced and the inexperienced surgeon will initially control bleeding with simple maneuvers, for instance, digital or manual compression of a bleeding artery. A less experienced surgeon will likely try to immediately perform direct suture or clamping of the vessel (type 1 decision), often without proper exposure, without obtaining proximal or distal control, and without taking the time to inform the team. However, the experienced surgeon will more likely pause and assess the available options, communicate with the anesthesiologist to check on the patient status, report the findings and the plan, allocate roles (improve lighting and exposure) and gather more resources (all type 2 decisions).

Simulation-based learning, by recreating “under test conditions, phenomena that are likely to occur in actual performance” (16) is particularly helpful in demonstrating the value of the decision-making process. When exposed to a critical scenario under artificial “classroom” conditions, i.e., a clinical case discussion, participants will rarely fail and will indicate the correct course of action. This means that type 2 decisions are mostly followed. However, if exposed to the same clinical scenario under simulated conditions, many more type 1 decisions are likely to occur. In the author’s opinion, this is one of the most important advantages of simulation-based training in trauma management.

However, not all type 1 decisions are necessarily wrong. For example, simple airway maneuvers, such as chin lift or jaw thrust (with restriction of cervical spine motion), or compression of external bleeding, are safe and can be expeditiously performed by a relatively inexperienced practitioner without much mental effort, and without incurring in patient hazards. However, key interventions, such as obtaining a definitive airway, decompressing a hemo- or pneumothorax, starting blood transfusion and taking the patient to OR, all fraught with complications and risks to the patient, should have the benefit of a pondered decision.

While training and experience may attenuate the trend toward repetitive type 1 use, a helpful tool is the use of intra-resuscitation or intraoperative time-outs (11, 17). These allow the entire team to reassess the situation and share concerns. A pause before action, in essence.

Another tool is the training of crisis containment strategies. One of these is the identification of the “surprise and startle” reaction. This technique has been developed for the training of airline crews in dealing with severe, unexpected inflight events, and is potentially transferable to the OR scenario. Airline pilots are trained to clearly identify the event and the response. They should avoid precipitous, type 1 decisions, and are trained to respond in a protocol manner, the “Stop-Aviate-Navigate-Communicate” protocol. The same can be trained for trauma teams (18), whereby a stop procedure and focus on the basics (ABC’s for the ER team, TBCS for the OR team). By identifying the event as a surprise, team members will force a stop, redirect the focus and weigh the options.

### Leadership

There is ample evidence to support the clear designation of a trauma team leader in the ER (19, 20). Although most trauma team leaders are surgeons (14), emergency physicians can also take up this role (21). However, much more relevant than the specialty *per se*, the attributes of a good trauma team leader are enabling input from the team members and using concise communication (22). Experienced trauma team leaders are associated with reduced time for resuscitation and for decision-making (23).

In the trauma OR, leadership is ideally a shared one. While the indication for surgery, i.e., the decision to operate, rests on the surgical team leader, the actual conduct of the operation requires shared decisions with the anesthesiologist. This is clearly demonstrated by the fact that most indications for damage control strategy (arterial pH and lactate, temperature, coagulopathy) (24), as well as the response to resuscitation, are physiologic variables easily obtained and updated by the anesthesiology team. Thus, a shared leadership can ideally take into account both what is happening in the surgical field and how is the patient’s physiology.

While the technical conduct is in the decision sphere of the surgeon, it is desirable that the anesthesiologist assumes a leadership role at critical moments, particularly during induction, or during severe unexpected events, such as cardiocirculatory arrest. For instance, a sudden intraoperative hemodynamic collapse may only be managed with cross-clamping of the aorta, and this may have to be indicated by the anesthesiologist. Another time for this reallocation of leadership is when the surgeon’s focus is too narrow to allow for a comprehensive view of the case, for instance during a procedure requiring focus, such as placing an intra-arterial shunt. Yet another example is during direct heart compressions, through a thoracotomy. The surgeon’s focus is directed at making sure the field is dry, the aortic clamp is not slipping and on the efficiency of the bimanual compressions. Thus, at this stage the anesthesiologist should assume a leadership role, deciding on drugs and timing of internal paddle defibrillation. Ego issues should not interfere with the patient management, as the most important person in the OR is, and always will be, the patient. As usual, good cooperation and excellent communication between surgeon and anesthesiologist is mandatory for this “two-headed” brain to work (25).

Good leadership means more that accomplishing the team’s mission – a live patient with the most severe injuries treated and with significant physiological reserve to recover in intensive care. Team leaders should also assess how the team members performed, how they felt and how can the team improve. This is discussed in more detail below (“Debriefing the team” section).

### Communication

As is obvious from the previous sections, communication is, by far, the most important NTS. Good communication is a team’s greatest asset, or its greatest drawback. In fact, it is estimated that 70 to 80% of healthcare errors are due to poor communication (26). Fortunately, there are rules for proper communication in emergency scenarios.

Both peri-resuscitation and perioperative communication should use the principles of closed-loop communication, which are:

• Direct communication, using name
o Both prearrival ED team and preoperative OR team briefings should serve to know names and allocate roles
• Visual contact, if possible.
o An exception to this is in the intraoperative setting, as the surgeon may not be able to do this if the surgical field requires attention.
• The recipient acknowledges the message and confirms that the message was clearly understood, and the task completed

**Figure 3:**
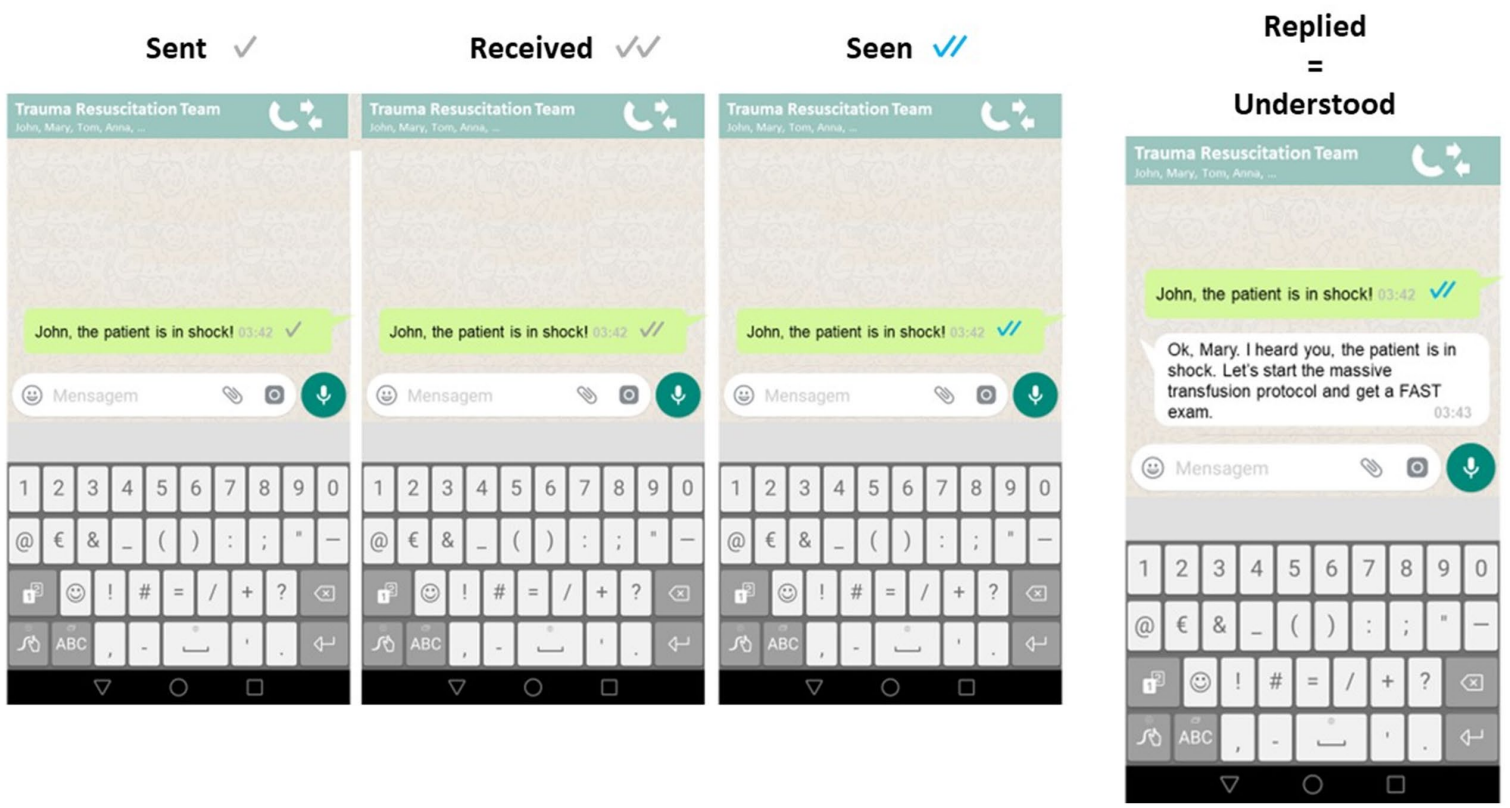
Clear symbology for sent, received and seen messages in WhatsApp. However, only when the receiver replies, does the sender really acknowledge that the message has been read and understood. This is translatable to communication in the trauma ER and OR.

Closed loop communication is associated with increased speed and efficiency of tasks in the resuscitation setting (27). The authors compare closed-loop communication to communication using the WhatsApp instant message platform, where there is a clear symbology for a sent, received and seen message. However, only when the sender receives the reply is a message truly understood (Figure 3).

Communication starts well before the operation starts, is mandatory during the operation, and is essential after the operation is concluded. Nonetheless, there should be safeguards to avoid over communication. A good rule of thumb is to see communication as a drug or as surgical instrument, i.e., it should be used in the proper timing and dosing. Interrupting the anesthesiologist during a difficult airway, or the surgeon during a difficult supraceliac aortic clamping can easily disrupt the focus. During these moments, only game changing information should be provided, such as sudden patient deterioration, or severe, unexpected intraoperative events. Again, this follows the strategy of the sterile cockpit rule of aviation, whereby during critical times only relevant information needs to be shared (28).

### Perioperative communication – the six-step approach

The authors’ group has developed a framework for communication in trauma management (25) – the six-step approach to perioperative communication in trauma. This stems from the recognition that there are key moments in the trauma patient’s path through the immediate preoperative, intraoperative and immediate postoperative periods, that mandate that the whole team is coordinated. Relevant data such as the patient’s physiology, suspected injuries, needed resources, and potential hazards should be shared.

This can be achieved with a communication strategy consisting of six distinct stages (Table 1):

**Table 1:** Six-step approach to perioperative communication in trauma surgery.

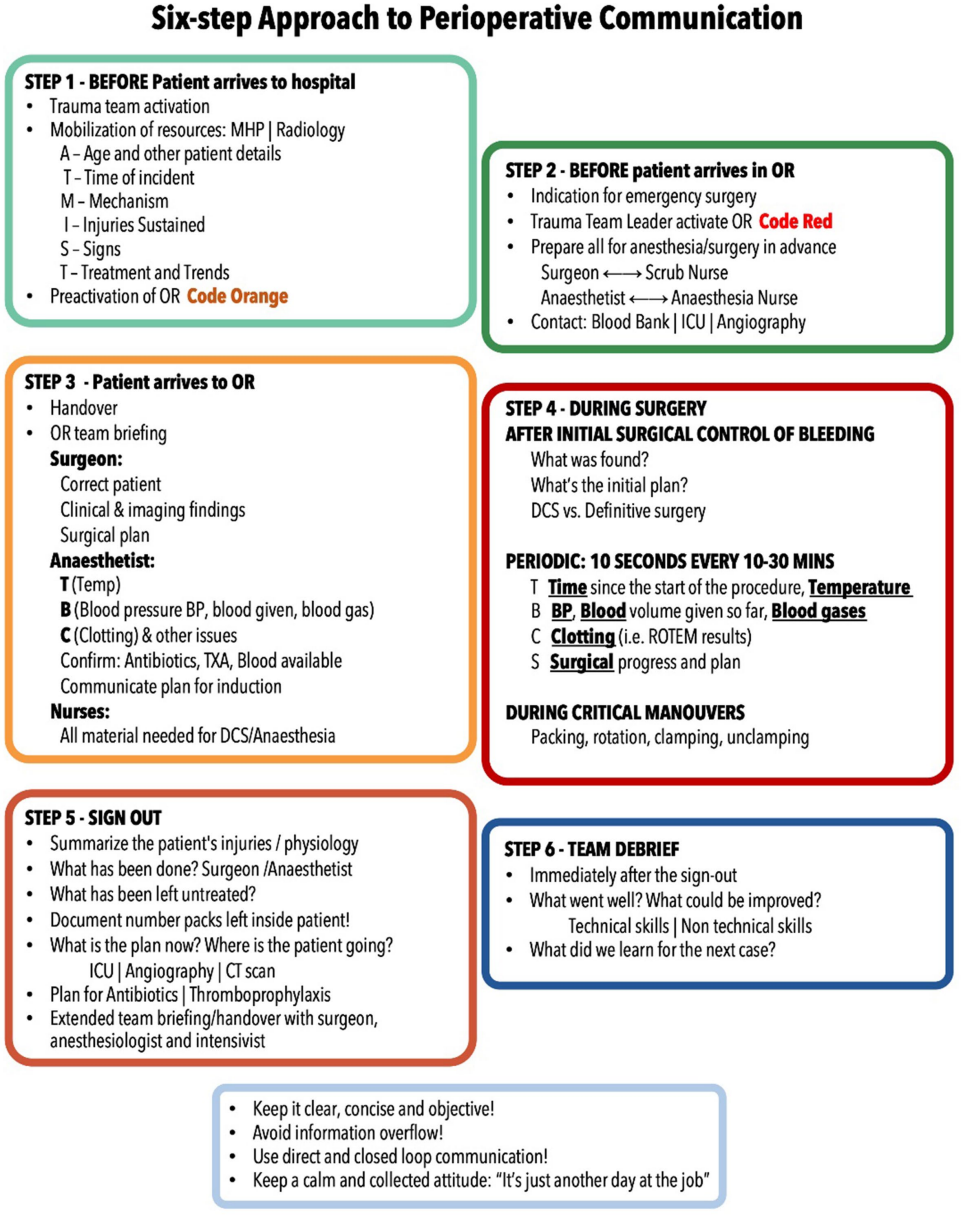

*Step 1 – Before patient arrival* – OR team notice

- A prehospital notification of the ER of a severely injured patient should prompt immediate trauma team activation and mobilization of resources (for instance, radiology, massive hemorrhage protocol, as well as preparation of the OR for a damage control procedure).
- Although precise information is rarely obtained at this stage, several key data from the AT-MIST should alert the OR team to ensure surgical material and anesthetic equipment are ready.
- In one of the authors’ institution, activation of the OR is designated by a specific “Code Orange.” This clearly informs the OR staff that a trauma patient will be arriving in the ER and may require emergency surgery. An operating theatre is booked with a team on standby until further notice from the team leader.
- At this stage it is desirable that both surgeon and anesthesiologist incorporate the ER trauma team; if not, step 3 becomes even more relevant.

*Step 2 – After patient arrival in ER* – Decision and preparation for emergency surgery

- Decision for emergency surgery should be swiftly communicated to the remaining OR team, including OR nurses, as well as assistant surgeons and anesthetic team (if not already present in the ER trauma team). This ensures that everything is ready to receive the patient in the operating room.
- Again, in one of the author’s institution a clear indication for emergency surgery is indicated by a warning of a “Code Red” to the OR team. The trauma team leader makes clear to the OR staff that the patient is immediately moving to the OR.
- While this turns a potential activation into a real one, the preliminary steps taken in Step 1 have made this stage easier.
- Surgical and anesthetic teams may take this time to brief the respective OR nursing teams (scrub and anesthetic) of specific requirements (for instance, thoracotomy tray, drugs). However, the authors advise that a trauma surgery protocol is the safest option.
- In some circumstances steps 1 and 2 are done together, for instance when patients arrive unannounced to the ER.

*Step 3 –Before surgery* – Preoperative communication

- This is a key moment in the OR and a potentially hazardous one. It starts with a handover of information from the ER team to the OR team, particularly if the anesthesiologist was not already present in the ER resuscitation. The OR nurses should be updated of the status, potential injuries and required resources. It is paramount that the OR nursing team be clearly allocated to positions - anesthesiology, scrub and circulating nurses – and quickly receive further information; all the team members’ names should be known.
- A key moment is anesthetic induction and there is real potential for patient deterioration. Loss of airway (“cannot intubate, cannot ventilate” scenario), tension pneumothorax and immediate cardiovascular collapse (due to loss of muscle tone and cardiovascular depression from anesthetic drugs in a shocked patient) require that anesthesiology and surgical teams coordinate their action. The surgical team should be scrubbed and gowned, and the patient prepped, before induction. Good role allocation and shared leadership are required, as well as optimal communication.
- Immediately before the incision, if time allows, there should be a brief pause in which the entire OR team agrees on the surgical plan, the patient’s clinical status, and to confirm that all materials, drugs, and blood products are available.

*Step 4 – During surgery* – Intraoperative communication.

- During surgery, immediate control of bleeding is the priority and once this is achieved the surgeon should request a short time-out. This will allow the team to pause, update status and reassess, avoiding spiraling into repeated type 1 decisions. This time out, using the TBCS tool, will be useful to grasp the physiology and the response to treatment, and plan the next move.
- A particularly hazardous moment is the opening of the peritoneum, which can cause loss of tamponade effect. Closed-loop communication is mandatory between operating surgeon and lead anesthesiologist at this stage.
- Regular intraoperative communication at intervals, again using the TBCS, will allow the team to “steer” the patient’s status more accurately; indeed, a patient initially planned to have a damage control procedure that recovers well with damage control resuscitation may be treated with definitive surgery.
- The decision to perform either a damage control or a definitive procedure should be clearly announced to the whole team.
- Communication should also be used during or, ideally, immediately after intraoperative adverse events, again using the TBCS.
- Intraoperative communication is also critical during key surgical manouvers that have significant physiologic impact, such as liver mobilization, major vessel clamping and unclamping, hepatic or renal hilar clamping, or heart manipulation. This requires closed-loop communication in order to properly coordinate the team.

*Step 5 – Sign Out* – Before patient leaving the OR

- This is another critical moment. Both the surgical (swab and instrument count, injuries found, procedures performed and timing of expected second-look) and anesthetic records (blood products, venous accesses, drugs, physiological status, past medical history from records) should be summarized and known by the respective surgical and anesthetic team leaders. The number of packs and the predicted timing of the second-look procedure should be clearly recorded and repeated by the team.
- The probabilities of other associated injuries being present should be addressed at this stage and the patient’s status reviewed, in order to decide between immediate transfer to CT (if not already done) or to the intensive care unit (ICU).
- Again, the TBCS tool is helpful to summarize what was done and what was the response.
- Contact with the ICU team, ideally started at step 2, is mandatory at this stage to update the patient’s status and define a plan (for instance, timing of second-look or definitive abdominal closure).
- The authors recommend that these trauma scenarios should be handled no differently from any other complex case which requires a multidisciplinary team meeting, such as an oncology patient. In fact, trauma patients can, by extent of injuries and physiological instability, be much more complex than many cancer patients. This trauma MDT is paramount for patient outcome.

*Stage 6 – Team debriefing*

- A trauma damage control operation can be an intense and at time off-putting experience, causing moral damage to the team members and potentially contributing to feelings of helplessness and burnout; this is particularly true when there was significant interpersonal tension or communication issues, or when the patient died in the OR. This requires a formal debriefing, which is explored in detail in the following section.

## Debriefing the team

Every trauma case is a learning opportunity, and debriefing is an invaluable tool to achieve this. However, it can be difficult to assemble the whole team after the trauma call or after the operation. There are several potential obstacles: reallocation to other clinical tasks, termination of shifts, lack of belief in debriefing and fear of accusation of misconduct. Nonetheless, it is desirable to gather the team, particularly after a challenging case, and conduct a formal debriefing. Good judgement is mandatory, as many team members may be on the defensive. In one of the author’s experience, a good way to prompt debriefing is to have the facilitator clearly state that every team member performed at the best of their individual skills and that the debriefing will only focus on the teamwork. This may help in removing some barriers to a frank discussion. Emphasizing that participation in the debriefing is voluntary, not mandatory, and that all shared information is confidential, is also desirable.

**Figure 4:**
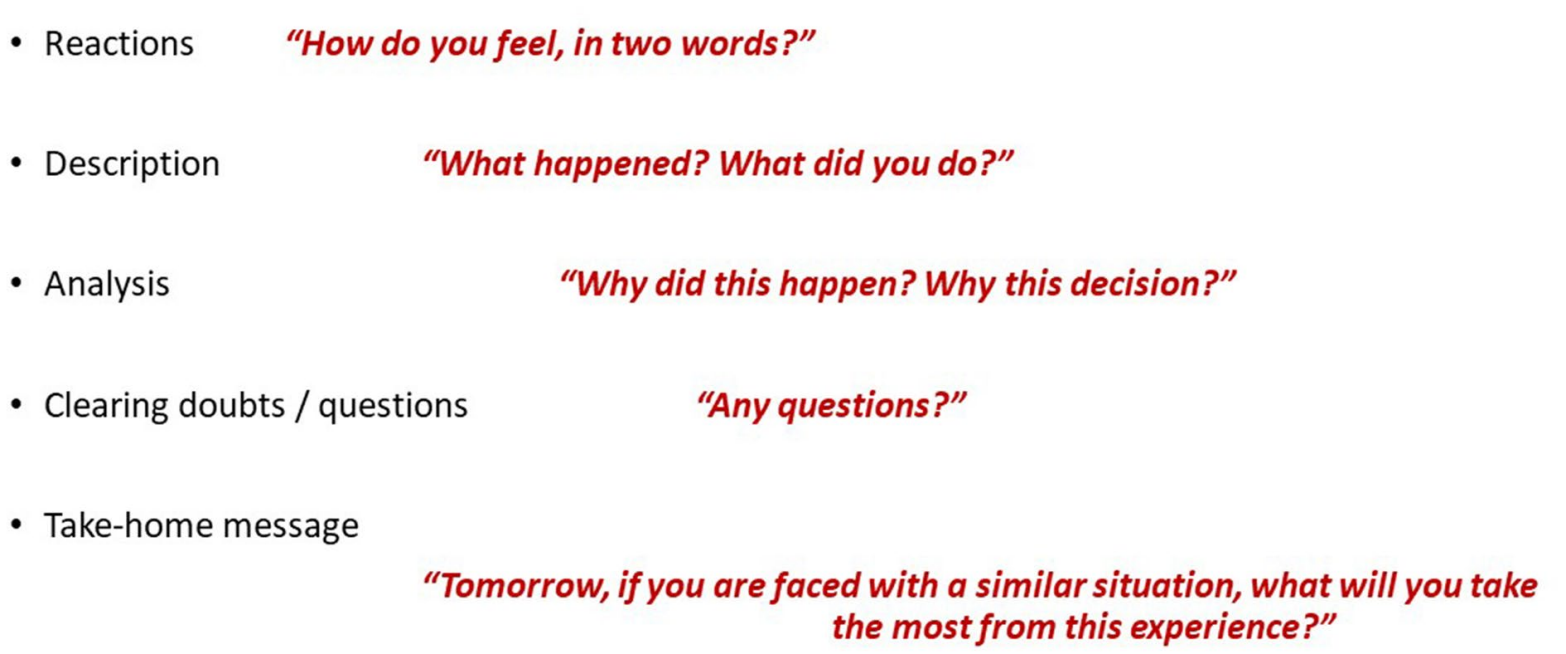
RDAT structure for post-resuscitation debriefing – Reactions/Description/Analysis/Questions?/Take-home message.

There are several strategies to conduct a postcritical debriefing. One such methods is the STOP – Summarize/Things that went well/Opportunities to improve/Points to action and responsibilities (29). When possible, the authors use a pedagogical structure for debriefing that can be used after simulation based training, the RDAT (Reactions, Description, Analysis, Questions? and Take-home message) (Figure 4) (30). With this method, the participants start by expressing feelings (Reactions phase) that can interfere with review of the facts (Description phase) and exploration (Analysis phase). After allowing for questions or doubts, the participants are invited to identify points for improvement (Take-home message).

In our experience the more junior the team member, the more they are willing to take part in the debriefing. The team leader should see this as a learning opportunity. Another purposed benefit of debriefing is the promotion of team cohesion (31), which is one of the key features of well-functioning teams (32).

In a worldwide snapshot of trauma team function and training, 15 and 23% of respondents reported that they performed debriefing always and often, respectively. A possible way to improve this is to promote debriefing practices in the undergraduate setting (see below). The authors recommend that team leaders should have formal training in debriefing techniques.

## Trauma team training focusing on non-technical skills

Trauma teams are not automatically formed simply by assembling a group of providers. Although human beings are social animals, the ability to function in a highly efficient team is not innate and requires attention to NTS. This has deserved increasing focus in recent years and there is compelling evidence to support the incorporation of NTS training into undergraduate and postgraduate settings, particularly in trauma. In fact, NTS has transcended trauma care and is spreading to other non-trauma surgical settings (33–35).

The European Trauma Course (ETC) was the first course to recognize this and specifically train NTS in the trauma setting (36). The ETC expanded on the training of clinical decision-making and technical skill acquisition promoted by the Advanced Trauma Life Support (ATLS) course. More recently, the ATLS program’s 10^th^ Edition has implemented teamwork training (37). For the authors, course instructors in both ATLS and ETC courses, rather than competing, the two courses add to each other. While ATLS provides the fundamentals of trauma care for the trauma team member, ETC promotes NTS for the trauma team members and team leader.

Regarding intraoperative teams, several notable courses, such as the NOTSS course (38), aim at improving patient safety in the perioperative setting. In the authors’ opinion, another way to achieve this is to include NTS in the already existing trauma courses. In a sense replicating in the OR setting what the ETC has done for the ER teams. The Definitive Surgical Trauma Care (DSTC), Definitive Anesthetic Trauma Care (DATC), Definitive Perioperative Nurses Trauma Care (DpNTC) courses are an excellent opportunity for this and allow the entire operating room team to participate in joint training sessions. Here the technical, decision-making and communication skills of surgeons, anesthesiologists and nurses are put to work in the immersive environment of a simulated damage control operating room.

Although the importance of NTS in trauma team function is well recognized, there is little real-world information on how teams are trained. In a recent survey, only 33% of hospitals provided trauma team training. Moreover, 60% of the trauma team members reported having had postgraduate training on NTS with only 24% at the undergraduate level (14). Regarding team training courses for the ER teams, the European Trauma Course was the most popular, immediately followed by local in-house courses. However, most trauma teams do not have the benefit of regular, simulation-based training.

## NTS teaching in undergraduate education

Undergraduate teaching aims to promote the acquisition of knowledge, skills and attitudes. However, emphasis has been mostly placed on individual, rather than teamwork skills. This undoubtedly produces well prepared junior doctors, but it is questionable whether these will successfully integrate clinical teams and become good team members, particularly in the emergency setting. Moreover, training for trauma teams is still rare from a global perspective, and even rarer in undergraduate education (14).

Fortunately, there is evidence that early training in NTS can not only lead to the acquisition, but also the retention, of NTS (39) and this has gained significant attention in recent years. Numerous studies have been conducted to explore the integration and effectiveness of teaching non-technical skills to medical students. Communication skills represent a cornerstone of non-technical competencies in Medicine. Studies have shown that enhanced communication skills lead to improved patient satisfaction, adherence to treatment, and even clinical outcomes. Simulation-based training and role-playing exercises have been shown to be effective in improving students’ ability to convey complex medical information in a patient-friendly manner (40).

Teamwork and leadership skills are also emphasized. Collaborative learning environments have been introduced in medical education, with group-based activities and interprofessional training to prepare future healthcare professionals for effective teamwork (41).

Moreover, promotion of debriefing habits is paramount in undergraduate education (42). The authors hope that the next generation of doctors has been trained in teamwork, is aware of the relevance of NTS and has gained the habit of debriefing the critical scenario.

## Future perspectives: hybrid room teams and NTS

In a typical clinical scenario, the trauma patient streamlines from prehospital handover to an ER team and, if surgical indication arises, to an OR team. Subsequently, the patient may undergo angioembolization in the interventional radiology (IR) suite. Such handovers are fraught with hazards and can contribute to deterioration of clinical practice (2). However, the development of hybrid rooms, with integrated resuscitation, imaging, surgical and angiography capacity, means that the same team can take care of the patient (43). This Trauma Hybrid Operating Room (THOR) concept not only mandates specific training in technical skills, but also poses significant challenges regarding NTS. Joining in the same room team elements with different skillsets (resuscitation, operating, imaging and endovascular) and backgrounds (ER, OR, IR) will require not only protocols and organization, but also a critical understanding of NTS from all participants. To the authors’ knowledge this has not been addressed specifically. As more and more institutions adopt the THOR concept, specific team training courses may arise for this particular context, incorporating NTS training alongside with technical skills training. The DSTC-DATC-DpNTC courses, given their flexibility and ability to incorporate add-on modules, could present an opportunity to provide team training for the very specific THOR context.

## Conclusion

Managing a severely injured trauma requires that every team member is at the best of his/her individual technical skills. But this is not enough, as outstanding individual work does not guarantee excellent team performance. Proficiency in non-technical skills, particularly the use of communication, are paramount to a good outcome. Fortunately, training opportunities are increasingly available, either with the ETC and inhouse courses, for ER teams; or with the joint DSApNTC courses, for OR teams. The inclusion of NTS in undergraduate curricula is a welcome step and will make the future doctors individually excellent, but also excellent team members and leaders. The future of trauma management will undoubtedly include hybrid rooms, and special attention should be given to training these teams in NTS.

## Author contributions

HA: Conceptualization, Data curation, Formal analysis, Funding acquisition, Methodology, Project administration, Supervision, Validation, Writing – original draft, Writing – review & editing. BM: Investigation, Writing – original draft, Writing – review & editing. LF: Validation, Writing – original draft, Writing – review & editing. SB: Conceptualization, Formal analysis, Funding acquisition, Investigation, Methodology, Supervision, Validation, Writing – original draft, Writing – review & editing.
